# Ultrasound-Visible 3D Nickel–Titanium Clip for Tumor Localization After Neoadjuvant Therapy in Early Breast Cancer: A Prospective Multicenter Real-World Study

**DOI:** 10.3390/diagnostics16172684

**Published:** 2026-08-22

**Authors:** Mattea Reinisch, Efstathia Cremer, Kilian Pankert, Diana Weber, Volker Hanf, Katja Engellandt, Sebastian Hentsch, Cordula Müller, Peter Dall, Matthias Losch, Petra Deuschle, Alexander Traut, Satyen Shenoy, Anita Engel, Dorothea Schindowski, Sherko Kuemmel, Simona Gipe

**Affiliations:** 1 Interdisciplinary Breast Unit, Kliniken Essen-Mitte, 45136 Essen, Germany; 2 Interdisciplinary Breast Unit, University Medical Center Mannheim, University of Heidelberg, 68167 Mannheim, Germany; 3 Department of Gynecology with Breast Center Charité-Universitätsmedizin Berlin, Corporate Member of Freie Universität Berlin and Humboldt-Universität zu Berlin, 10117 Berlin, Germany; 4 Center for Familial Breast and Ovarian Cancer, Institute for Hereditary Cancers, Center for Integrated Oncology (CIO), Faculty of Medicine and University Hospital Cologne, University of Cologne, 50937 Cologne, Germany; 5 Breast Unit, Diakonissen-Stiftungs-Krankenhaus Speyer, 67346 Speyer, Germany; 6 Breast Unit, St. Barbara Klinik Hamm-Heessen, 59073 Hamm, Germany; 7 Breast Unit, Klinikum Fürth, 90766 Fürth, Germany; 8 Breast Unit, Ev. Bethesda Krankenhaus Duisburg, 47053 Duisburg, Germany; 9 Breast Unit, Städtisches Klinikum Solingen, 42653 Solingen, Germany; 10 Breast Unit, Kreiskrankenhaus Bergstraße Heppenheim, 64646 Heppenheim, Germany; 11 Breast Unit, Städtisches Klinikum Lüneburg gGmbH, 21339 Lüneburg, Germany; 12 Breast Unit, Prosper-Hospital gGmbH, Kreis Recklinghausen, 45659 Recklinghausen, Germany; 13 Breast Unit, Marienhaus Klinikum Hetzelstift Neustadt, 67434 Neustadt an der Weinstrasse, Germany; 14 Diakonissenkrankenhaus Speyer, Medizinisches Versorgungszentrum Rhein-Haardt, Gynäkologie, 67346 Speyer, Germany

**Keywords:** early breast cancer, neoadjuvant systemic therapy, clip detection, ultrasound-guided localization, wire-guided localization, biopsy marker, mammographic verification, prospective registry

## Abstract

**Background:** Neoadjuvant systemic therapy (NST) enables tumor downsizing and increases the feasibility of breast-conserving surgery (BCS) in patients with early breast cancer (EBC). Reliable tumor localization after NST remains challenging, particularly in patients with a complete clinical response. If clips are not visible on ultrasound, stereotactic mammography-guided wire localization is required, which involves additional radiation exposure and may increase patient discomfort and procedural complexity. The 3D-shaped Tumark^®^ Vision clip may enable ultrasound-guided localization after NST. **Methods:** In this prospective multicenter registry study (NCT04468113), 324 patients with biopsy-proven EBC scheduled for NST and breast-conserving surgery were enrolled across 19 German centers. Clip placement was performed under ultrasound guidance prior to NST, and clip detectability was assessed at predefined time points during therapy (4–8, 9–12, and ≥13 weeks after treatment initiation). Detection rates, visualization, and preoperative localization methods were recorded. Non-detectable clips required stereotactic wire localization, whereas ultrasound-guided localization was performed for detectable clips. **Results:** The preoperative detection rate was 91.1%. Clip detectability was higher in patients with longer NST durations and partial response by imaging. Ultrasound-guided wire localization was feasible in 214 patients (83.9%); among these, 165 patients (64.7%) underwent localization without and 49 patients (19.2%) with post-procedural mammographic verification. Stereotactic localization was required in 41 patients (16.1%), primarily due to non-visualization of the clip on ultrasound. The accuracy of ultrasound for residual tumor assessment was moderate (72.0%), with a sensitivity of 70.7%, indicating limitations in its ability to reliably assess residual tumor extent as a standalone modality. **Conclusions:** The 3D nickel–titanium clip enables ultrasound-guided tumor localization after NST in the majority of patients with early breast cancer, although additional mammographic guidance remains necessary in a relevant proportion of cases.

## 1. Introduction

Neoadjuvant systemic therapy (NST) has become an integral component of treatment for patients with early breast cancer (EBC) [1]. In addition to increasing the rate of breast-conserving surgery through tumor downstaging, NST provides important prognostic information and guides the selection of post-neoadjuvant systemic therapy according to the pathological response. As modern systemic treatment regimens have substantially increased pathological complete response (pCR) rates, accurate localization of the original tumor site has become increasingly important to ensure complete surgical excision of the tumor bed and to guide subsequent treatment decisions [2].

Preoperative localization is essential for the surgical management of non-palpable breast lesions. Although several techniques are available, wire-guided localization (WGL) remains the most widely used approach because of its broad availability and relatively low cost [3,4]. The purpose of the wires is to enable clear identification of the tumor bed, which is the central tenet to this approach. This poses a challenge, especially in EBC patients with post-NST pathological complete response (pCR), i.e., EBC patients whose tumors have decreased in volume after NST to a point where it is difficult or even impossible to detect them by ultrasound or mammography. To counter this shortcoming, one widely used clinical approach is to mark the breast lesions with a commercially available clip prior to NST. Following NST and prior to surgery, these clips are visualized by either ultrasound or mammography and a wire is placed in the tissue surrounding the clip to identify the lesion. Reliable ultrasound detectability of the clip could reduce the need for stereotactic mammography-guided localization, thereby decreasing radiation exposure, improving patient comfort, and reducing procedural complexity and resource utilization.

The Tumark^®^ Vision clip (Somatex^®^ Medical Technologies, Berlin, Germany) is a sterile, preloaded disposable system for marking tissue areas and is a CE-approved medical device. Following deployment, the marker expands into a three-dimensional spherical configuration that provides immediate fixation within the tissue. This spherical shape, in combination with its echogenic surface structure, enables effective imaging with ultrasound. In an initial feasibility study including 50 patients with EBC, the Tumark Vision^®^ clips achieved a 100% detection rate immediately after placement, with no clip displacement observed during the first week after implantation [5].

Reliable ultrasound-guided localization of a previously marked tumor site is essential and offers several advantages over stereotactic guidance, including no radiation exposure, increased patient comfort, and reduced logistical and financial demands. Finding ways to improve preoperative management by avoiding radiographic wire localization is a persistent goal in breast surgery and has led to the approval of other marking methods, such as magnetic seeds. Nevertheless, the optimal biopsy marker for reliable ultrasound detection after tumor regression has not yet been identified. Conventional nickel–titanium clips remain attractive because they are inexpensive, widely available, MRI-compatible, and easily integrated into routine clinical workflows, with ubiquitous use globally. However, evidence regarding the influence of clip design on long-term ultrasound detectability after NST remains limited.

To address this evidence gap, we conducted a prospective multicenter registry trial, Ultra3Detect (Ultra3D) (NCT04468113). The primary objective was to evaluate the preoperative ultrasound detectability of the three-dimensional Tumark^®^ Vision clip in patients with early breast cancer undergoing neoadjuvant systemic therapy; secondary objectives included the assessment of clip detectability throughout NST, identification of factors associated with successful ultrasound detection, and evaluation of the potential to reduce the need for stereotactic mammography-guided localization in routine clinical practice.

## 2. Materials and Methods

### 2.1. Study Design

Ultra3Detect is a prospective, multicenter registry trial that was conducted at 19 certified breast cancer centers across Germany (NCT04468113).

Women aged ≥18 years with sonographically detectable uni- or bilateral intramammary biopsy-proven EBC with an indication for NST and BCS and without any distant metastasis or allergy to titanium/nickel were eligible for enrolment in the study. Detailed inclusion and exclusion criteria are presented in Table A1 (Appendix A). In general, patients presenting with sonographically suspicious breast lesions and scheduled for ultrasound-guided core needle biopsy with clip placement were screened for eligibility. The Tumark® Vision clips used were manufactured by SOMATEX Medical Technologies GmbH, Berlin, Germany. The manufacturer’s address at the time of the study approval in 2020 was Hohenzollerndamm 150/151, 14199 Berlin, Germany. Patients were enrolled only after histopathological confirmation of early breast cancer and confirmation of indication for NST.

Patients with a cT1 to cT3 tumor size were included, irrespective of the nodal status. Patients were included only if the biopsy results were positive for EBC with an indication for NST. Patients with extensive microcalcifications requiring stereotactic localization were excluded because the study specifically evaluated ultrasound-guided clip detection.

Data management and analysis were conducted at the Kliniken Essen-Mitte, Essen, Germany. The trial was conducted in accordance the Declaration of Helsinki and was approved by local institutional ethical committee (North Rhine Medical Association, protocol code 2020004, 28 January 2020). All patients provided written informed consent prior to entering the trial.

### 2.2. Study Procedure and Data Collection

**Figure 1:**
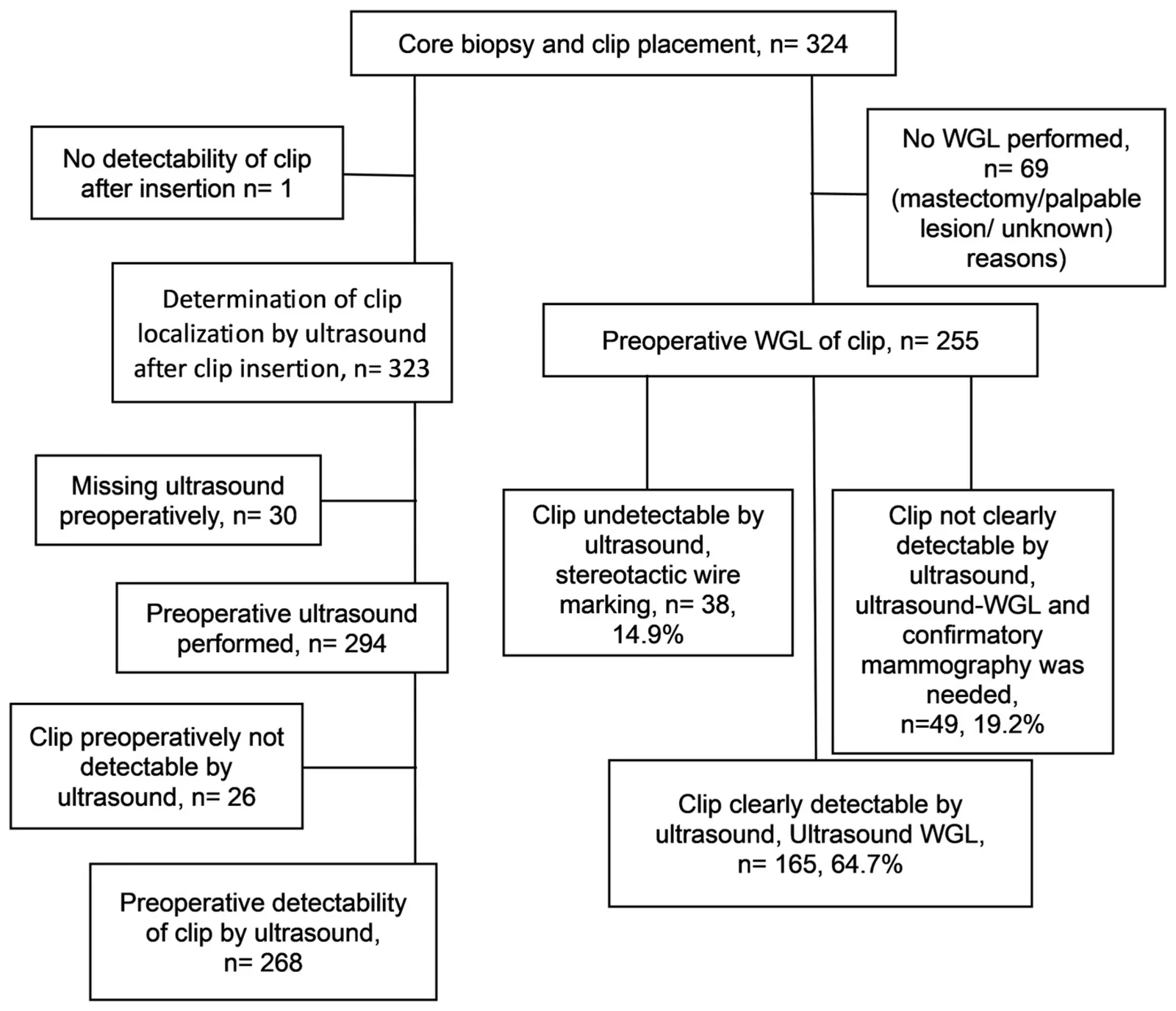
CONSORT diagram of the Ultra3D study; WGL: wired-guided localization.

The study design (patient flow) of the Ultra3Detect study is given in Figure 1. All patients were scheduled to receive NST, and ultrasound examinations were scheduled at predefined time points during this treatment (4–8, 9–12, and ≥13 weeks after treatment initiation) and immediately before surgery. The predefined primary endpoint was the preoperative ultrasound detection rate of the clip following completion of NST. Ultrasound examinations were performed at predefined time points during treatment to evaluate clip detectability over time. The timing of these assessments reflected routine clinical practice within the participating German breast cancer centers. A clip was considered detectable when it could be unequivocally identified by ultrasound at the expected tumor bed location. If the clip was clearly detectable on preoperative ultrasound, ultrasound-guided wire localization was performed; if ultrasound detection was not possible, stereotactic mammography-guided localization was used. In cases of uncertain ultrasound visualization, ultrasound-guided wire localization was followed by mammographic confirmation of correct wire placement before surgery. Device-related complications, such as hematoma, and imaging-related issues, such as artefact formation, were prospectively recorded. Clip migration was not a predefined study endpoint and was therefore not systematically assessed or quantified, as previous evidence suggests that clinically relevant migration of correctly deployed breast markers is uncommon [5].

### 2.3. Statistical Analysis

#### 2.3.1. Sample Size Calculation

Because Ultra3Detect was designed as a prospective observational registry without a comparative study arm, no formal hypothesis-driven sample size was calculated. The planned sample size of approximately 300 patients was based on feasibility considerations and the expectation that approximately one-third of patients would achieve pathological complete response following NST. This was considered sufficient to provide a robust descriptive assessment of clip detectability in clinically relevant subgroups.

#### 2.3.2. Endpoints and Subgroup Analysis

The primary endpoint was the preoperative ultrasound detection rate of the biopsy marker following completion of NST, and secondary exploratory endpoints included clip detectability during NST, localization technique, clinicopathological predictors of successful clip detection, and concordance between clinical and pathological staging.

Descriptive statistics were employed to evaluate data gathered in the Ultra3Detect study. Demographics and other basic data, including disease characteristics, are listed and summarized descriptively. Categorical data are presented as frequencies and percentages, and for continuous data, the mean, standard deviation, median, minimum, and maximum were calculated. Missing values, which occurred due to the multicenter design and variability in data collection across centers, were excluded from percentage calculations; no data imputation was performed.

The cohort of patients in whom the tumor was marked with a clip are described using absolute numbers and relative proportions. A two-sample *t*-test or a Wilcoxon rank sum test were used to assess differences in continuous variables between groups, while Fisher’s exact test was used to assess differences between groups for categorical variables. The primary endpoint of the trial was the detection rate (DR) of the clip by ultrasound prior to surgery following NST. The sonographic clip DR was calculated as the number of patients with successful sonographic clip detection divided by the total number of patients in whom sonographic clip detection was attempted. In addition to the point estimate, 95% binomial confidence intervals (CIs) were also calculated.

The effects of different predictor factors on the sonographic DR were examined using multivariable logistic regression analysis. All tests were two-sided and *p*-values < 0.05 were considered statistically significant. Analyses were performed with IBM SPSS Statistics (Version 23; IBM Corp., Armonk, NY, USA).

## 3. Results

### 3.1. Patient Demographics

A total of 339 patients from 19 certified breast cancer centers across Germany were enrolled in the study. Complete data were available for 324 patients, who were included in the final analysis. The details of patient disposition and baseline characteristics are given in Figure 1 and Table 1, respectively.

Enrolled patients had a median (range) age of 56 (25–86) years; presented with unilateral EBC, mostly in the upper two quadrants; and had a median (range) body mass index of 26 (16.9–52.7) kg/m2 and breast density of class ACR b (*n* = 122, 43.1%) or ACR c (*n* = 123, 43.5%).

The clinical staging of EBC by ultrasound at the time of clipping was cT1c (*n* = 136, 42.1%), cT2 (*n* = 163, 50.1%) for tumor status, and cN0 (*n* = 242, 74.8%) for nodal status. Tumors were mostly of the NST subtype (*n* = 302, 93.2%) and of Grade 2 (*n*= 113, 34.7%) or Grade 3 (*n* = 208, 64.4%). The proportion of patients with HER2+ or TNBC disease was 37.8% and 36.8%, respectively.

Patients underwent NST for a median (range) of 20 (6–32) weeks. A total of 177 patients (74.1%) received an anthracycline-containing chemotherapy regimen; targeted therapy with trastuzumab/pertuzumab was applied in 32.1% of all included patients (Table 1). In 20 cases (6.2%), the NST type was not reported.

### 3.2. Tumark Vision^®^ Clip Detection

#### 3.2.1. Observations During Clipping and Clip Safety

**Figure 2:**
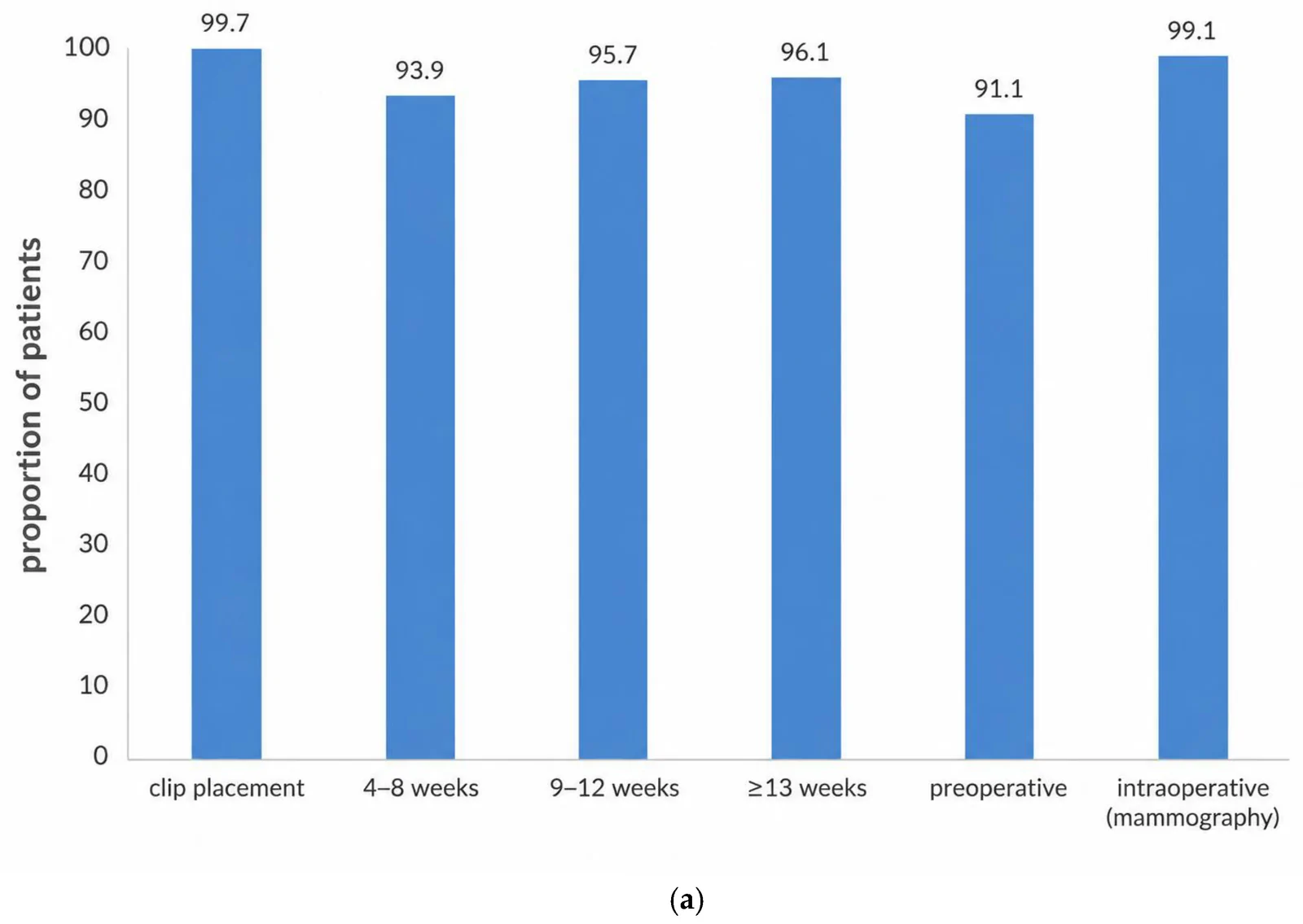

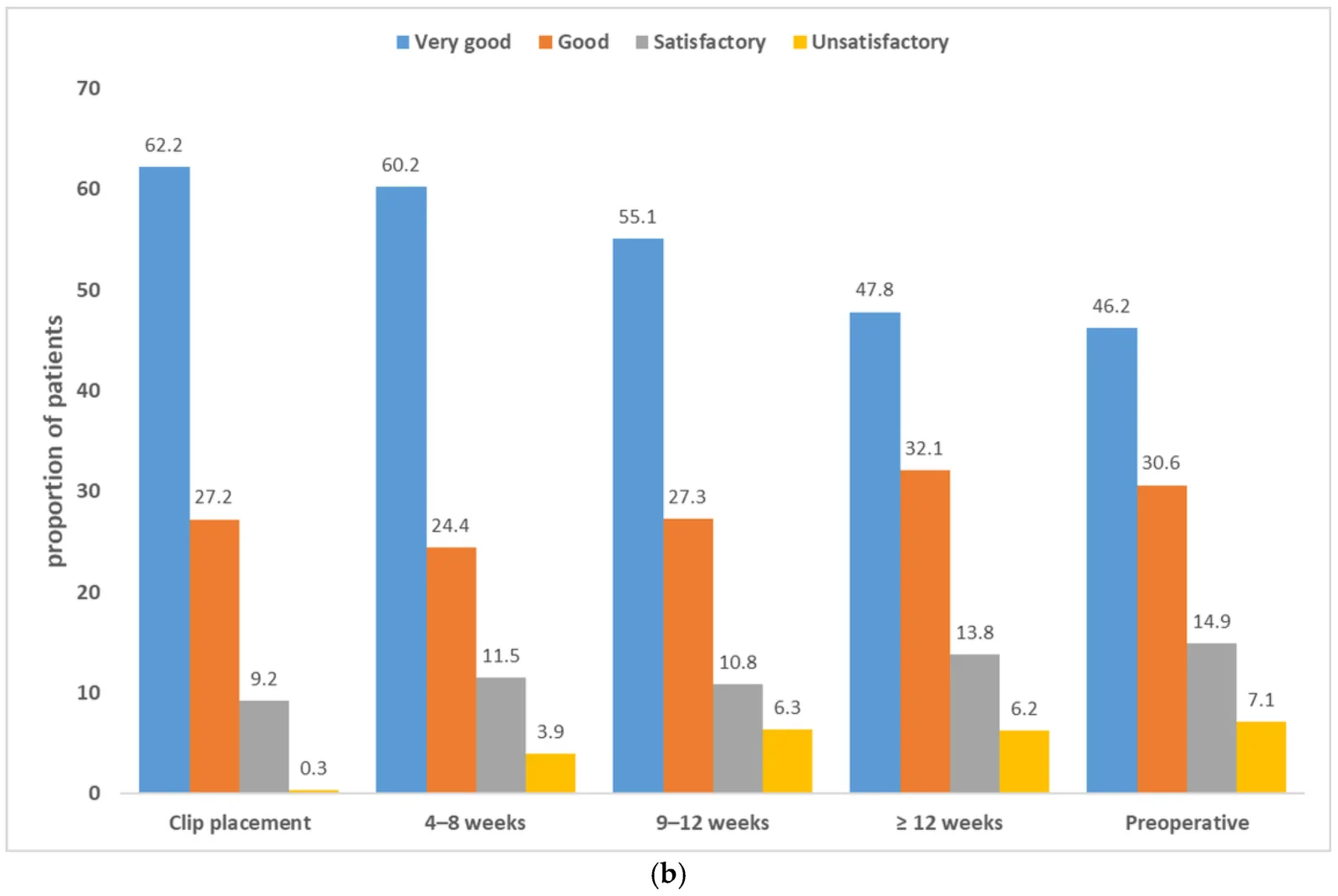
(**a**) Ultrasound clip detectability from implantation to operation. (**b**) Ease of ultrasound clip detectability from implantation to pre-operative stage.

Clip placement under ultrasound guidance was successful in 321 of 324 patients (99.1%). In three patients (0.9%), a second clip had to be inserted due to technical difficulties during the initial placement. Immediately after implantation, the clip was clearly detectable by ultrasound in 323 patients (99.7%). Investigators rated clip visibility as “very good” or “good” in 92.6% of cases (*n* = 301, 92.6%; Figure 2b), and in 93.2% (*n* = 302) of patients, the clip was observed to be placed directly in the tumor bed. Overall, the procedure was well tolerated with a low complication rate. Three cases of post-interventional hematoma (0.9%) were reported. In addition, three cases of clip-related imaging artefacts were documented, including posterior acoustic shadowing and intratumoral mirror artefacts. No infections or allergic reactions and no clinically evident clip migration was reported within the collected dataset.

#### 3.2.2. Observations During NST

Ultrasound detectability remained consistently high throughout NST. The DR of the inserted clip by ultrasound after 4–8 weeks (*N* = 282), 9–12 weeks (*N* = 222) and ≥13 weeks (*N* = 224) of NST was 93.9%, 95.7%, and 96.1%, respectively (Figure 2a).

Although overall detectability remained high, the subjective assessment of clip visibility gradually shifted from “very good” to “good” or “satisfactory”, reflecting progressive tumor shrinkage during treatment (Figure 2b).

#### 3.2.3. Pre-Operative Observations

The predefined primary endpoint was the preoperative ultrasound detection rate following completion of NST. Overall, the clip was successfully detected by ultrasound in 268 of 294 patients, corresponding to a detection rate of 91.1% (Figure 2a), and in 206 patients (76.8%), clip visibility was rated as “very good” or “good” (Figure 2b). Detection rates were comparable across tumor subtypes.

#### 3.2.4. Pre-Operative Wire-Guided Localization

Among patients undergoing wire-guided localization, ultrasound guidance alone was sufficient in 165 cases (64.7%). In an additional 49 patients (19.2%), ultrasound-guided localization was performed with mammographic confirmation of correct wire placement because clip visualization was considered uncertain. Stereotactic localization was required in 38 patients (14.9%) (Figure 1). The rate of successful ultrasound-guided wire localization without the need for confirmatory mammography was comparable between patients with pathological complete remission (pCR) and those with non-pCR (53.2% vs. 50.0%, respectively).

### 3.3. Concurrence in Clinical Staging

The clinical staging of patients before and after NST and after surgery is presented in Table 2.

Following NST, 158 patients (49.8%) were classified as ycT0, while 152 patients (46.9%) achieved ypT0. Ultrasound therefore slightly overestimated complete tumor response. A similar pattern was observed for nodal staging, with ycN0 reported in 95.6% of patients compared with a pathological ypN0 rate of 86.7%.

**Figure 3:**
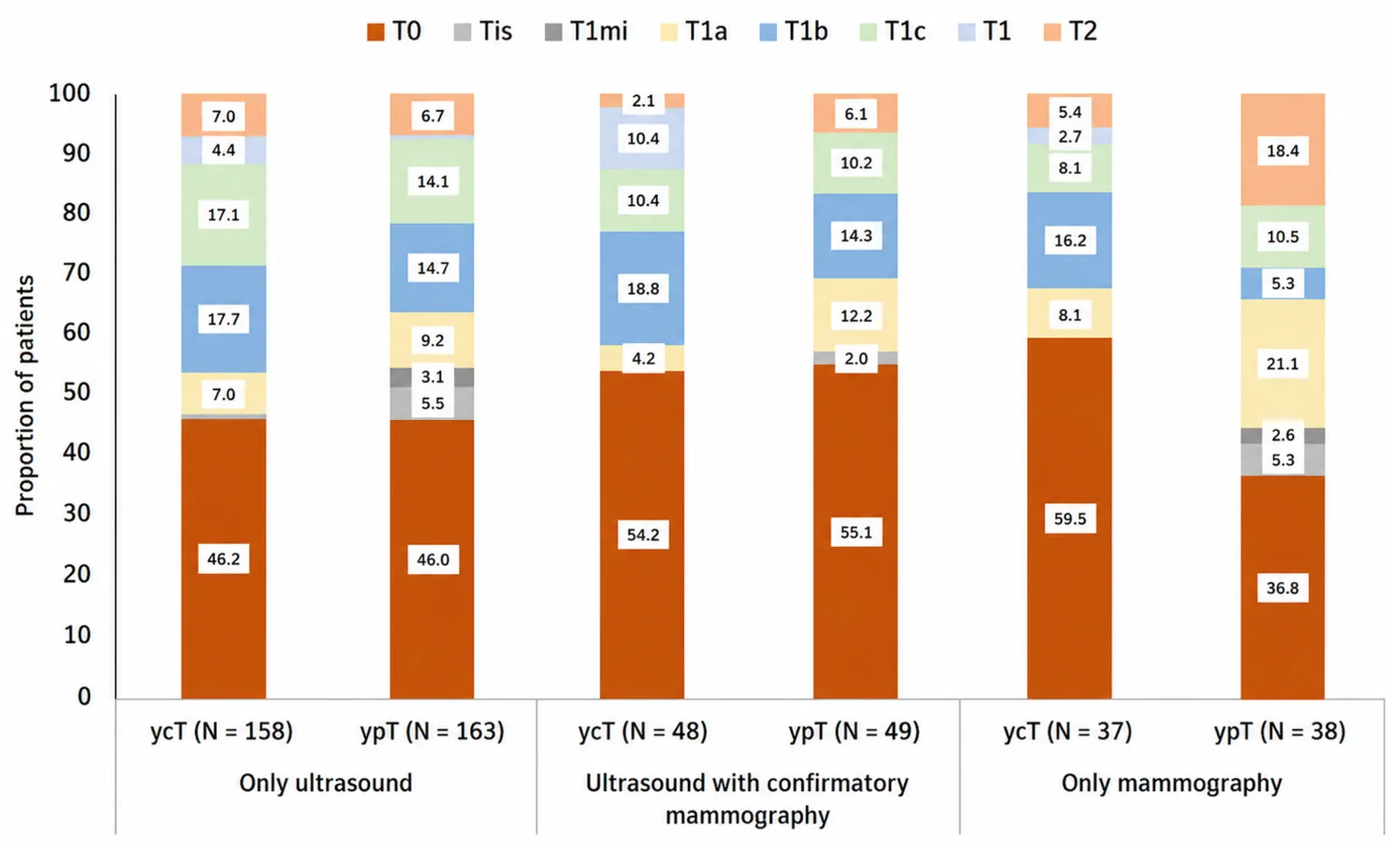
Concurrence between clinical staging after NST and pathological staging after surgery according to the technique used for clip detection; cT = clinical stage, pT = pathological stage.

A comparison between post-NST clinical staging and post-surgery pathological staging using the clip detection method is presented in Figure 3.

Overall, concordance between post-treatment clinical staging and final pathology was highest in patients in whom the clip could be clearly identified by ultrasound. In contrast, there was a higher frequency of pathological upstaging in patients requiring mammographic localization.

Overall, ultrasound demonstrated a moderate accuracy of 72.01% for predicting residual disease, with a sensitivity of 70.73% (95% CI: 63.13% to 77.57%) and a specificity of 73.38% (95% CI: 65.66% to 80.17%). The positive predictive value of 73.9% and negative predictive value of 70.2% indicate a moderate accuracy of sonographic response assessment. These findings reflect the limitations of ultrasound in evaluating residual tumor burden rather than limitations related to clip detectability. Therefore, ultrasound assessment of residual tumor burden should be interpreted independently from clip visibility.

### 3.4. Factors Associated with Clip Detectability After NST

**Figure 4:**
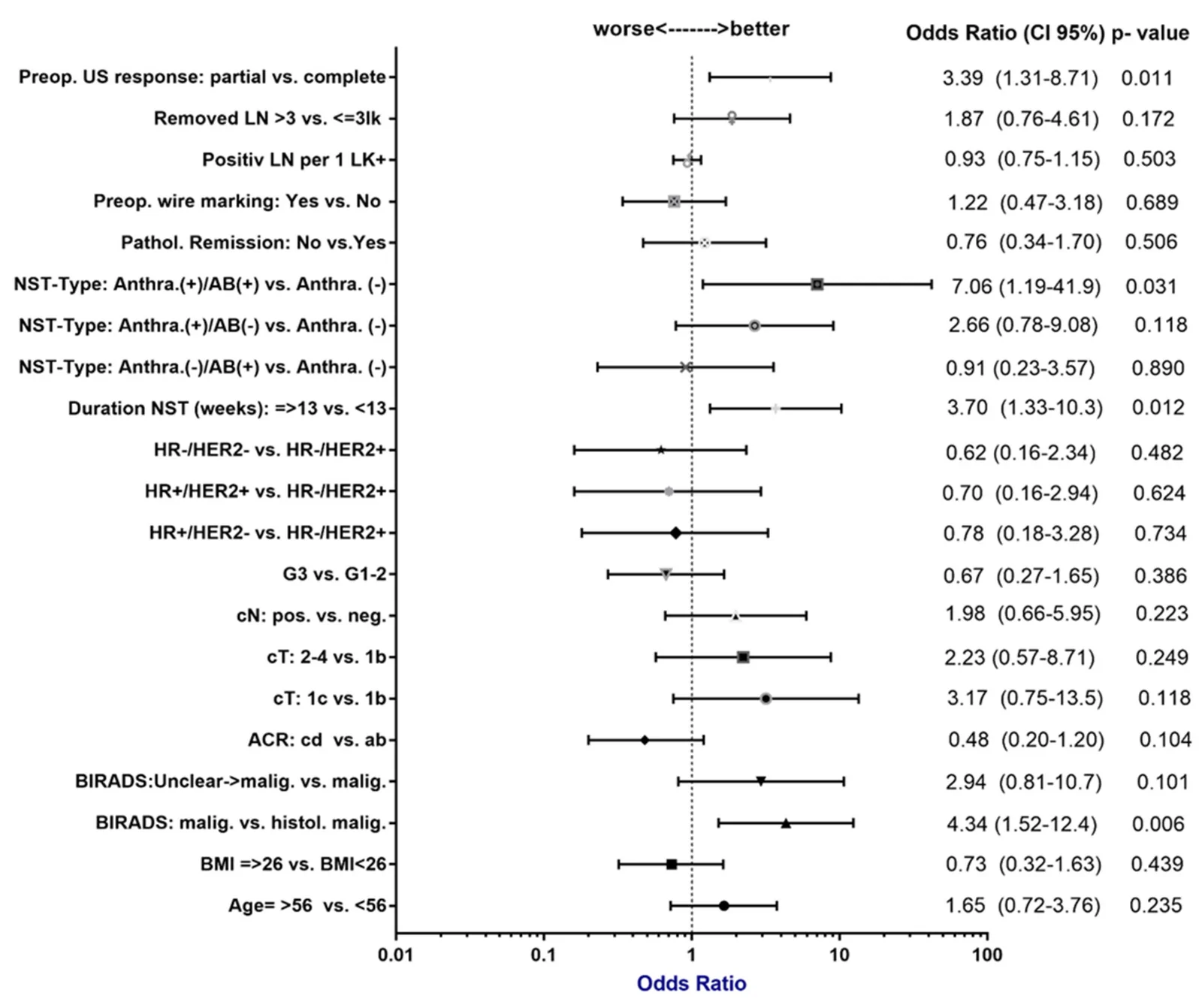
Forest plot of factors associated with sonographic detectability of the clip prior to surgery; US: ultrasound; LN: lymph nodes; NST: neoadjuvant systemic therapy; Anthra: anthracycline; AB: anti-Her2 antibody; HR: hormone receptor; ACR: American College of Radiology; BMI: body mass index.

Of the 294 patients who underwent preoperative ultrasound, the clip was detectable in 268 (91.2%). Several factors associated with clip detectability were assessed in a multivariable logistic regression analysis (Figure 4, Table 3). Improved detectability was significantly associated with a neoadjuvant chemotherapy duration of ≥13 weeks (*p* = 0.012), anthracycline-based chemotherapy combined with dual HER2 blockade (*p* = 0.031), and a partial response on ultrasound imaging (*p* = 0.011) (Figure 4, Table 3). No significant associations were observed for patient age, body mass index, breast density, tumor subtype, tumor size, or nodal status; importantly, pathological complete response (pCR) was also not associated with clip detectability (Table 3).

## 4. Discussion

The DR of clips in breast cancer after NST remains a pivotal element in surgical planning for successful lesion excision. Wire-guided localization has traditionally been the standard approach due to its wide availability, low cost, and proven effectiveness. However, mammographic guidance may require additional imaging, resulting in increased radiation exposure and greater procedural complexity.

This prospective, multicenter Ultra3Detect study aimed to systematically evaluate the ultrasound visibility of the Tumark^®^ Vision clip for localizing breast cancer lesions following NST and provide valuable insights into current DRs in real-word settings. The primary endpoint of the trial was the detection rate of the clip by ultrasound prior to surgery following NST. To the authors’ best knowledge, this is the largest cohort in studies analyzing this parameter. The detection rate reported by the investigator was 91.1% prior to surgery. Due to tumor shrinkage under NST and the change in echogenicity of the breast tissue, the certainty of clip visibility by ultrasound changed over time during therapy. A total of 93.2% of physicians reported a “very good” clip visibility prior to NST directly after clip placement; this decreased to 76.8% after chemotherapy prior to surgery. Nevertheless, ultrasound-guided wire localization was performed using ultrasound alone in only 64.7% of cases. In 49 patients (19.2%), mammography was needed to confirm the correct placement of the wire, but none needed correction. The clip DR by ultrasound was lower in patients with complete clinical response compared to those with residual disease prior to surgery. Overall, the Tumark^®^ Vision clip demonstrated a high detection rate (91.1%). Ultrasound-guided wire localization was feasible in 83.9% of patients, whereas stereotactic localization was required 16.1%. Clip implantation and subsequent localization procedures showed a favorable safety profile, with no serious device-related complications and no clinically evident clip migration reported.

In multivariate analysis, higher clip detectability was independently associated with longer treatment duration, anthracycline-based chemotherapy combined with dual HER2 blockade, and partial clinical response. The latter observation is biologically plausible, as residual tumor tissue may facilitate sonographic identification of the biopsy marker compared with identification in patients achieving complete clinical response.

Our findings are in line with published data [6,7], which have reported detection rates of around 72.5% to 91%. The high DR of more than 90% is most likely due to the three-dimensional shapes of the clip used by our team, also used by Minella et al. [6], as well as the continuous improvements in ultrasound technology and image quality in recent years. Despite these encouraging results, ultrasound detection was not feasible in all patients. Consequently, a proportion of patients still required stereotactic mammography-guided localization, highlighting the need for further improvements in localization strategies. To overcome these limitations, several wire-free localization technologies have been developed.

Probe-guided localization (PGL) using devices such as the LOCalizer^®^ or radiofrequency identification (RFID) tags has emerged as a promising alternative to WGL. PGL involves inserting a magnetic or RFID marker into the lesion, which can be localized intraoperatively using a handheld probe without the need for wires. The literature reports success rates exceeding 90% for PGL in post-NST settings, with the same DR as wire-guided localization techniques [8,9,10,11]. The accuracy of RFID tag placement is comparable to that of wire localization; however, its higher costs may limit its widespread adoption as an alternative to WGL [12]. These methods offer wire-free localization alternatives that may enhance patient comfort and provide greater flexibility in clinical workflow. A prospective multicenter observational study evaluated different marking techniques for target lymph nodes in node-positive breast cancer treated with neoadjuvant therapy. Probe-guided detection methods demonstrated higher detection rates compared with clip marking; however, clip-based localization achieved an overall detection rate of 89.6% [13]. These findings indicate that clip-based localization remains feasible even in the anatomically challenging axillary setting. In our study, the Tumark^®^ Vision clip demonstrated a detection rate of 91.1% for breast tumor localization after NST, supporting the feasibility of ultrasound-based clip detection in clinical practice.

Despite their advantages, probe-guided technologies are associated with several practical limitations. MRI susceptibility artefacts caused by RFID tags or magnetic seeds may impair assessments of residual disease after NST [14]. This potentially limits their utility in patient populations undergoing NST, where MRI surveillance is needed for monitoring treatment response. Furthermore, the accuracy of the detection probe may be affected by tissue density, tag migration, or interference from surrounding metallic implants. In the event of a non-retrieved or ‘lost’ tag, the retention of the device in the breast tissue presents a significant challenge for future diagnostic imaging, as persistent MRI susceptibility artefacts may complicate interpretation of future breast MRI examinations [8,14]. Reported re-excision rates of approximately 8% have also been described for RFID-guided localization by Munday et al. [12]. Additionally, the cost and availability of RFID or magnetic guidance systems limit widespread adoption. A study by Pete et al. [11] noted that while PGL has a higher patient satisfaction profile, there was a slight increase in the need for intraoperative adjustments compared to WGL, particularly in cases with tag migration or poor marker placement.

While PGL technology is promising, the optimal localization approach requires further evaluation in larger comparative studies. The prospective MELODY study (NCT05559411), including 7416 patients from 30 countries, is expected to provide important data on oncological safety, re-excision rates, and patient-reported outcomes for different wire-free localization technologies [15]. However, re-excision rates should not be considered a direct measure of clip performance, as they are influenced by multiple factors beyond marker localization, including tumor biology, response to neoadjuvant therapy, surgical technique, and institutional margin assessment strategies.

An alternative to expensive probe detection devices and WGL may be the addition of artificial intelligence (AI) to a standard ultrasound procedure in order to detect the clip reliably in the breast tissue at any time point during the treatment journey. There are efforts to develop an algorithm that can be implemented in a standard ultrasound device to mark clips in dense tissue in real time on an ultrasound image, despite bad visibility [16]. The wire can then be safely placed in the AI-marked area in a comparable manner to the standard procedure in which the clip is viewed in a standard ultrasound image. This AI-based software has been investigated with different clip fabrics but is currently not yet approved.

Importantly, the ability to detect the clip does not reflect the accuracy of ultrasound in assessing residual tumor burden. While the clip itself remained detectable in more than 90% of patients, ultrasound demonstrated only moderate accuracy (72.0%) for predicting residual disease. This finding reflects the well-recognized limitations of ultrasound for response assessment after NST rather than the limitations of the biopsy marker itself. Consequently, clip detectability and imaging accuracy represent two distinct clinical concepts and should be interpreted separately. This limitation emphasizes the importance of combining ultrasound findings with other clinical and imaging parameters for optimal response assessment and the need for improved diagnostic approaches.

Some limitations of the Ultra3Detect study should be considered. First, as a prospective registry, there is neither a control arm nor a randomized setting; therefore, the reported detection rate (91.1%) primarily reflects the feasibility and performance of this localization approach in a real-world multicenter setting. Although the results can be compared with previously published data, the absence of a comparator group limits conclusions regarding the relative clinical advantages of ultrasound-visible clip localization compared with established localization techniques.

Second, ultrasound assessments were performed across multiple certified breast cancer centers following standardized national protocols, which aim to ensure consistent quality. However, inter-observer variability was not formally assessed. Given the operator-dependent nature of ultrasound, this may influence the generalizability of the findings, but also reflects the real-world evidence.

Third, detailed information on individual operator experience was not available due to the multicenter design. However, all participating sites were certified German breast cancer centers subject to strict quality assurance standards. These include minimum annual case volumes and the involvement of experienced senior breast surgeons in clinical care. These standards aim to ensure a consistently high level of expertise in both surgical and ultrasound-based procedures.

Fourth, patient-reported outcomes and procedure-related satisfaction were not measured, as the primary objective of the registry was the technical evaluation of clip detectability.

Fifth, although initial clip placement was highly accurate, clip migration during NST was not a predefined endpoint of the Ultra3Detect study and was therefore not systematically quantified. However, clip visibility and position were assessed during scheduled ultrasound follow-up examinations, and no clinically relevant migration was reported.

Finally, the observational study design does not allow conclusions regarding the superiority of the Tumark^®^ Vision clip compared with other biopsy markers. As all participating centers used the same three-dimensional ultrasound-visible clip, the findings are specific to this marker type and cannot be directly extrapolated to other marker designs. Comparative studies evaluating different marker systems are required to determine whether differences in marker design influence ultrasound visibility and localization performance.

Ultimately, the choice of localization technique should be tailored to the individual patient characteristics, lesion properties, and institutional resources [17,18]. Consideration of economic costs, breast esthetic outcomes, and lesion characteristics (number, location, and breast volume) is essential, and the integration of artificial intelligence and machine learning with existing localization technologies could lead to adaptive surgical navigation systems. The localization method should be carefully selected considering size, location, resources, radiologist, and surgeon.

Recent studies suggest that a combined approach, including early clip placement and the use of intraoperative ultrasound or RFID-based detection, may improve localization success [8,11,19]. For example, intraoperative ultrasound guided by RFID detection has demonstrated a DR comparable to traditional WGL but with improved patient experience and operational efficiency. Nevertheless, the cost-effectiveness and accessibility of RFID technology remain a concern, potentially limiting widespread adoption.

Compared with stereotactic techniques, ultrasound-guided localization offers several practical advantages. These include the avoidance of ionizing radiation, as well as improved patient comfort due to the less invasive and more flexible nature of the procedure. In addition, ultrasound-guided approaches may facilitate clinical workflow, as they can often be performed directly by the treating surgical team without dependence on radiology scheduling [20]. Unlike many wire-free devices that require additional specialized equipment, ultrasound-visible clips can be localized using standard ultrasound equipment available in breast centers.

While a formal cost-effectiveness analysis was beyond the scope of this study, a study by Konen et al. [21] suggests that ultrasound-guided localization may contribute to increased efficiency and reduced resource utilization. This may be particularly relevant in resource-limited settings or high-volume centers. A retrospective review using hematoma-directed ultrasound-guided lumpectomy have demonstrated potential cost savings by avoiding additional localization procedures in selected patients [22]. In contrast, permanent ultrasound-visible markers may offer advantages in patients undergoing neoadjuvant therapy, where the original tumor may become difficult to identify after treatment response. Importantly, in our study, similar rates of successful ultrasound-guided localization without additional mammographic confirmation suggest that clip-based ultrasound localization is feasible even in patients achieving pCR.

## 5. Conclusions

This study is the first prospective investigation into the visibility of a three-dimensional clip in a large cohort of patients with early breast cancer following NST. Using this clip can help reduce the dependence on stereotactic wire localization, thereby decreasing hospital workload, radiation exposure, and patient discomfort.

While the use of ultrasound-visible clips can reduce reliance on stereotactic localization, they do not eliminate the need for mammography-guided procedures. A pragmatic, multimodal approach remains essential to ensure reliable localization after NST.

Additionally, because wire marking is relatively inexpensive compared to more advanced detection methods, this approach is especially beneficial in resource-limited settings or where access to costly technology is restricted.

## Figures and Tables

**Table 1:** Patient characteristics.

	*N* = 324
	*n* (%)
General	
Age, median (range)	56 (25–86)
BMI, median (range) White	26 (17–53) 324 (100)
Tumor characteristics	
Tumor subtype	
No Special Type (NST)	302 (93.2)
Lobular	7 (2.1)
Other *	11 (3.5)
Missing	4 (1.2)
Tumor grade	
G1	2 (0.6)
G2	113 (34.7)
G3	208 (64.4)
Gx	1 (0.3)
Tumor receptor subtype	
HR+/HER2+	73 (22.5)
HR+/HER2−	82 (25.2)
HR−/HER2+	50 (15.3)
HR−/HER2−	119 (36.8)
Clinical tumor stage	
cT1a	0
cT1b	18 (5.5)
cT1c	136 (42.1)
cT2	163 (50.1)
cT3	7 (2.1)
Tumor size in mm, median (range)	21 (6–70)
ACR classification	
ACR a	17 (6.0)
ACR b	122 (43.1)
ACR c	123 (43.5)
ACR d	20 (7.4)
Missing	42
Clinical nodal stage	
cN0	243 (74.8)
cN+ ^#^	82 (25.2)
Number of weeks on NST, median (range)	20 (6–32)

* Includes metaplastic, apocrine, neuroendocrine, and non-definable subtypes. ^#^ Nodal stage prior to NST was captured as “positive” versus “negative”. Note—Missing values are not included in calculation of percentages. ACR, American College of Radiology; BMI, body mass index, HER2, human epidermal growth factor receptor 2; HR, hormone receptor; NST, neoadjuvant systemic therapy.

**Table 2:** Clinical TNM classification pre-NST, post-NST, and post-surgery.

	Pre-NST *N* (%)	Post-NST *N* (%)	Post-Surgery *N* (%)
Overall Clinical tumor stage	(cT) 324	(ycT) 324	(ypT) 324
T0	0	158 (49.8)	152 (46.9)
Tis	-	1 (0.3)	17 (5.2)
T1mi	-	0	7 (2.2)
T1a	0	17 (5.4)	32 (9.9)
T1b	18 (5.5)	55 (17.3)	35 (10.8)
T1c	136 (42.1)	44 (13.9)	42 (13.0)
T1 (undifferentiable)	-	15 (4.7)	1 (0.3)
T2	163 (50.1)	26 (8.2)	35 (10.8)
T3	7 (2.1)	1 (0.3)	3 (0.9)
T4	0	0	0
Missing	0	7	0
Clinical nodal stage	(cN)	(ycN)	(ypN)
cN0	243 (74.8)	303 (95.6)	281 (86.7)
cN1a	cN+ 82 (25.2) *	11 (3.5) *	30 (9.2)
cN2a			10 (3.1)
cN3a			2 (0.6)
Nx	0	3 (1.0)	1 (0.3)
Missing	0	7	0
Clinical metastatic stage			
M0	324 (100.0)		321 (99.1)
M1	0		3 (0.9)

* Nodal stage prior to NST (neoadjuvant systemic therapy) was classified as “positive” or “negative”.

**Table 3:** Correlation of various parameters with sonographic clip detectability (*n* = 294).

Variable	Total *n* (%)	Endpoint *n* (%)	Odds Ratio (95% CI)	*p*-Value
Total	294 (100%)	268 (91.1%)		
Age				
Per 1 year			1.01 (0.98–1.05)	0.482
<56	148 (50.3%)	132 (89.2%)	1 (ref.)	
≥56	146 (49.7%)	136 (93.2%)	1.65 (0.72–3.76)	0.235
BMI				
Per 1 unit			0.97 (0.90–1.05)	0.477
<26	157 (53.4%)	145 (92.4%)	1 (ref.)	
≥26	137 (46.6%)	123 (89.8%)	0.73 (0.32–1.63)	0.439
BI-RADS				
BIRADS 6	100 (34.0%)	85 (85.0%)	1 (ref.)	
BIRADS 5	128 (43.5%)	123 (96.1%)	4.34 (1.52–12.4)	0.006
BIRADS 4	53 (18.0%)	50 (94.3%)	2.94 (0.81–10.7)	0.101
Other	11 (3.7%)	8 (72.7%)	0.47 (0.11–1.98)	0.304
Missing	2 (0.7%)	2 (100%)	–	–
ACR				
a–b	127 (43.2%)	119 (93.7%)	1 (ref.)	
c–d	130 (44.2%)	114 (87.7%)	0.48 (0.20–1.20)	0.104
X	37 (12.6%)	35 (94.6%)	1.18 (0.24–5.80)	0.809
cT				
T1b	16 (5.4%)	13 (81.3%)	1 (ref.)	
T1c	118 (40.1%)	110 (93.2%)	3.17 (0.75–13.5)	0.118
T2–4	160 (54.4%)	145 (90.6%)	2.23 (0.57–8.71)	0.249
Per 1 cm			0.77 (0.55–1.08)	0.134
cN				
Negative	219 (74.5%)	197 (90.0%)	1 (ref.)	
Positive	75 (25.5%)	71 (94.7%)	1.98 (0.66–5.95)	0.223
Grading				
G1–2	102 (34.7%)	95 (93.1%)	1 (ref.)	
G3	192 (65.3%)	173 (90.1%)	0.67 (0.27–1.65)	0.386
Tumor Subtype				
HR−/HER2+	46 (15.6%)	43 (93.5%)	1 (ref.)	
HR+/HER2−	73 (24.8%)	67 (91.8%)	0.78 (0.18–3.28)	0.734
HR+/HER2+	66 (22.4%)	60 (90.9%)	0.70 (0.16–2.94)	0.624
HR−/HER2−	109 (37.1%)	98 (89.9%)	0.62 (0.16–2.34)	0.482
NST Duration per week			1.07 (0.98–1.17)	0.109
<13 weeks	27 (9.2%)	21 (77.8%)	1 (ref.)	
≥13 weeks	265 (90.8%)	246 (92.0%)	3.70 (1.33–10.3)	0.012
NST Type				
Anthracycline-free, no antibody	21 (7.1%)	17 (81.0%)	1 (ref.)	
Anthracycline-free + antibody	34 (11.6%)	27 (79.4%)	0.91 (0.23–3.57)	0.890
Anthracycline-based, no antibody	160 (54.4%)	147 (91.9%)	2.66 (0.78–9.08)	0.118
Anthracycline-based + antibody	62 (21.1%)	60 (96.8%)	7.06 (1.19–41.9)	0.031
Unknown	17 (5.8%)	17 (100%)	–	–
Pathologic Complete Remission				
Yes (T0/Tis/N0)	154 (52.4%)	142 (92.2%)	1 (ref.)	
No	140 (47.6%)	126 (90.0%)	0.76 (0.34–1.70)	0.506
Positive LNs (per 1LN pos.)			0.93 (0.75–1.15)	0.503
Removed LNs (per 1 LN)			1.07 (0.95–1.25)	0.262
≤3 LN	177 (60.4%)	158 (89.3%)	1 (ref.)	
>3 LN	116 (39.6%)	109 (94.0%)	1.87 (0.76–4.61)	0.172
Preoperative Ultrasound Response				
Complete	147 (50.0%)	127 (86.4%)	1 (ref.)	
Partial	135 (45.9%)	129 (95.6%)	3.39 (1.32–8.71)	0.011
Stable	7 (2.4%)	7 (100%)	–	–
Progressive	4 (1.4%)	4 (100%)	–	–
Missing	1 (0.3%)	1 (100%)	–	–

LN: lymph node; NST: neoadjuvant systemic therapy; HR: hormone receptor; ACR: American College of Radiology; BMI: body mass index.

## Data Availability

The data presented in this study are available from the corresponding author upon reasonable request. The data are not publicly available due to privacy and ethical restrictions.

## Abbreviations

The following abbreviations are used in this manuscript:

NST	neoadjuvant systemic therapy
BCS	breast-conserving therapy
EBC	early breast cancer
pCR	pathological complete response
MRI	magnetic resonance imaging
WGL	wire-guided localization
DR	detection rate
ACR	American College of Radiology
LN	lymph node
US	ultrasound
BMI	body mass index
AB	anti-Her2 antibody
HR	hormone receptor
PGL	probe-guided localization
RFID	radiofrequency identification
AI	artificial intelligence

## Appendix A

**Table A1:** Inclusion and exclusion criteria for the Ultra3D study.

Inclusion criteria: ▪ Female aged ≥18 years; ▪ Provided signed informed consent; ▪ Provided consent to NST; ▪ A suspicious uni/bilateral intramammary focus safely identified by USG; ▪ No evidence of distant metastasis; ▪ Indicated for BCS ▪ No prior clip placement in confirmed intramammary carcinoma; ▪ The capacity to undergo NST; ▪ High compliance with planned surgical interventions; ▪ The ability to understand the scope of the Ultra3D study.
Exclusion criteria ▪ Allergy to titanium and/or nickel; ▪ Pregnant; ▪ Ipsilateral relapse; ▪ A history of prior extensive breast surgery; ▪ Inflammatory breast cancer; ▪ Extramammary breast cancer; ▪ Multicentric or multifocal breast cancer; ▪ Inoperable breast cancer; ▪ Already undergoing adjuvant/neoadjuvant therapy; ▪ Lack of ability to understand the scope of the Ultra3D study.

BCS, breast conservation surgery; NST, neoadjuvant therapy; USG; ultrasonography.

## References

[1] Colomer R., Saura C., Sánchez-Rovira P., Pascual T., Rubio I.T., Burgués O., Marcos L., Rodríguez C.A., Martín M., Lluch A. (2019). Neoadjuvant Management of Early Breast Cancer: A Clinical and Investigational Position Statement. Oncologist.

[2] Park-Simon T.W., Müller V., Albert U.S., Banys-Paluchowski M., Bartsch R., Bauerfeind I., Bjelic-Radisic V., Blohmer J.-U., Dall P., Ditsch N. (2025). AGO Recommendations for the Diagnosis and Treatment of Patients with Early Breast Cancer: Update 2025. Breast Care.

[3] Shah A.D., Mehta A.K., Talati N., Brem R., Margolies L.R. (2018). Breast tissue markers: Why? What’s out there? How do I choose?. Clin. Imaging.

[4] Banys-Paluchowski M., Kühn T., Masannat Y., Rubio I., de Boniface J., Ditsch N., Cakmak G.K., Karakatsanis A., Dave R., Hahn M. (2023). Localization Techniques for Non-Palpable Breast Lesions: Current Status, Knowledge Gaps, and Rationale for the MELODY Study (EUBREAST-4/iBRA-NET, NCT 05559411). Cancers.

[5] Rüland A.M., Hagemann F., Reinisch M., Holtschmidt J., Kümmel A., Dittmer-Grabowski C., Stöblen F., Rotthaus H., Dreesmann V., Blohmer J.-U. (2018). Using a New Marker Clip System in Breast Cancer: Tumark Vision® Clip—Feasibility Testing in Everyday Clinical Practice. Breast Care.

[6] Minella C., Villasco A., D’alonzo M., Cellini L., Accomasso F., Actis S., Biglia N. (2022). Surgery after Neoadjuvant Chemotherapy: A Clip-Based Technique to Improve Surgical Outcomes, a Single-Center Experience. Cancers.

[7] Koo J.H., Kim E.-K., Moon H.J., Yoon J.H., Park V.Y., Kim M.J. (2019). Comparison of breast tissue markers for tumor localization in breast cancer patients undergoing neoadjuvant chemotherapy. Ultrasonography.

[8] Lowes S., Bell A., Milligan R., Amonkar S., Leaver A. (2020). Use of Hologic LOCalizer radiofrequency identification (RFID) tags to localise impalpable breast lesions and axillary nodes: Experience of the first 150 cases in a UK breast unit. Clin. Radiol..

[9] Webster A.J.B., Kelly B.N.B., McGugin C., Coopey S.B.M., Smith B.L.M., A Gadd M., Specht M.C.M. (2022). Comparison of Wireless Localization Alternatives with Wire Localization for Nonpalpable Breast Lesions. J. Am. Coll. Surg..

[10] McGugin C., Spivey T., Coopey S., Smith B., Kelly B., Gadd M., Hughes K., Dontchos B., Specht M. (2019). Radiofrequency identification tag localization is comparable to wire localization for non-palpable breast lesions. Breast Cancer Res. Treat..

[11] Pete R., Pinard C., Sirodot F., Molnar I., Dressaire M., Ginzac A., Abrial C., Durando X., Tekath M. (2025). Patient satisfaction with radio-frequency identification (RFID) tag localization compared with wire localization for nonpalpable breast lesions: The RFID trial. BMC Cancer.

[12] Munday C., Malhotra A., Taif S., Omotade A., Menon A., Mokbel K. (2025). Evaluation of Hologic LOCalizer™ RFID Tags for Preoperative Localization of Breast Lesions: A Single-Center Experience. Diagnostics.

[13] Banys-Paluchowski M., Hartmann S., de Boniface J., Gentilini O.D., Ditsch N., Stickeler E., Cakmak G.K., Hauptmann M., Schroth J., Thill M. (2026). Marking Techniques for Target Lymph Nodes in Node-Positive Breast Cancer Treated with Neoadjuvant Therapy in the AXSANA/EUBREAST-03/AGO-B-053 Study. J. Clin. Oncol..

[14] Lamb L.R., Gilman L., Specht M., D’ALessandro H.A., Miles R.C., Lehman C.D. (2021). Retrospective review of preoperative radiofrequency tag localization of breast lesions in 848 patients. Am. J. Roentgenol..

[15] Banys-Paluchowski M., Kuehn T., Masannat Y., Ditsch N., Cabioglu N., Harvey J., Harvey J., Pankratjevaite L., Murawa D., Gasparri M.L. (2024). MELODY: A prospective non-interventional multicenter cohort study to evaluate different imaging-guided methods for localization of malignant breast lesions (EUBREAST-4/iBRA-NET). J. Clin. Oncol..

[16] Kodarapu R., Fassbender T., Reinisch M., Sudikatus P., Kümmel J.R.A.S. (2023). DP23 DEEP-LEARNING-BASED LOCALIZATION OF BREAST BIOPSY MARKER IN ULTRASOUND DATA. Abstracts of the 12th International Charité Mayo Conference on “Global Perspectives and Future Directions in Women’s Cancer”. Anticancer Res..

[17] Norman C., Lafaurie G., Uhercik M., Kasem A., Sinha P. (2021). Novel wire-free techniques for localization of impalpable breast lesions—A review of current options. Breast J..

[18] Chopra S., Khosla M., Vidya R. (2023). Innovations and Challenges in Breast Cancer Care: A Review. Medicina.

[19] Kumar S., Kadow B.B., Lamkin M.K. (2011). Challenges with the introduction of radio-frequency identification systems into a manufacturer’s supply chain—A pilot study. Enterp. Inf. Syst..

[20] Cheung B.H.H., Co M., Lui T.T.N., Kwong A. (2024). Evolution of localization methods for non-palpable breast lesions: A literature review from a translational medicine perspective. Transl. Breast Cancer Res..

[21] Konen J., Murphy S., Berkman A., Ahern T.P., Sowden M. (2020). Intraoperative ultrasound guidance with an ultrasound-visible clip: A practical and cost-effective option for breast cancer localization. J. Ultrasound Med..

[22] Merrill A.Y., Ochoa D., Klimberg V.S., Hill E.L., Preston M., Neisler K., Henry-Tillman R.S. (2018). Cutting healthcare costs with hematoma-directed ultrasound-guided breast lumpectomy. Ann. Surg. Oncol..

